# Circulating Concentrations of Key Regulators of Nitric Oxide Production in Undernourished Sheep Carrying Single and Multiple Fetuses

**DOI:** 10.3390/ani10010065

**Published:** 2019-12-30

**Authors:** Fiammetta Berlinguer, Cristian Porcu, Giovanni Molle, Andrea Cabiddu, Maria Dattena, Marilia Gallus, Valeria Pasciu, Sara Succu, Francesca D. Sotgiu, Panagiotis Paliogiannis, Salvatore Sotgia, Arduino A. Mangoni, Antonio Gonzalez-Bulnes, Ciriaco Carru, Angelo Zinellu

**Affiliations:** 1 Department of Veterinary Medicine, University of Sassari, Via Vienna 2, 07100 Sassari, Italy; porcu.cristian@gmail.com (C.P.); vpasciu@uniss.it (V.P.); succus@uniss.it (S.S.); fran.sotgiu@gmail.com (F.D.S.); 2 AGRIS Sardegna, Loc. Bonassai, 07100 Sassari, Italy; gmolle@agrisricerca.it (G.M.); acabiddu@agrisricerca.it (A.C.); mdattena@agrisricerca.it (M.D.); mgallus@agrisricerca.it (M.G.); 3 Department of Biomedical Sciences, University of Sassari, Viale S. Pietro, 43/C, 07100 Sassari, Italy; panospaliogiannis@gmail.com (P.P.); ssotgia@uniss.it (S.S.); carru@uniss.it (C.C.); azinellu@uniss.it (A.Z.); 4 Department of Clinical Pharmacology, College of Medicine and Public Health, Flinders University, Adelaide 5042, Australia; arduino.mangoni@flinders.edu.au; 5 Comparative Physiology Group, SGIT-INIA, Av. Puerta de Hierro, 18, 28040 Madrid, Spain; bulnes@inia.es

**Keywords:** ADMA, SDMA, L-homoarginine, sheep, twins, triplets, L-arginine, nitric oxide

## Abstract

**Simple Summary:**

The present study aimed to determine the blood concentrations of L-arginine, asymmetric dimethylarginine (ADMA), symmetric dimethylarginine (SDMA), and L-homoarginine, modulating nitric oxide (NO) synthesis, in single, twin. and triplet pregnancies in ewes undergoing either dietary energy restriction or receiving 100% of their energy requirements. Blood concentrations of L-arginine, of its metabolites. and the ratio between NO synthesis boosters and inhibitors are altered in energy-restricted ewes, these alterations being higher in ewes carrying multiple fetuses.

**Abstract:**

The aim of this study was to investigate the blood concentrations of L-arginine, asymmetric dimethylarginine (ADMA), symmetric dimethylarginine (SDMA), and L-homoarginine, which are regulators of nitric oxide (NO) synthesis, in single, twin, and triplet pregnancies in ewes undergoing either a dietary energy restriction or receiving 100% of their energy requirements. From day 24 to 100 of pregnancy, the ewes were fed ryegrass hay and two different iso-proteic concentrates fulfilling either 100% of ewes’ energy requirements (control group; *n* = 30, 14 singleton pregnancies, 12 twin pregnancies, and 4 triplet pregnancies) or only 45% (feed-restricted group; *n* = 29; 11 singleton pregnancies, 15 twin pregnancies, and 3 triplet pregnancies). Blood samples were collected monthly to measure, by capillary electrophoresis, the circulating concentrations of arginine, ADMA, homoarginine, SDMA, and of other amino acids not involved in NO synthesis to rule out possible direct effects of diet restriction on their concentrations. No differences between groups were observed in the circulating concentrations of most of the amino acids investigated. L-homoarginine increased markedly in both groups during pregnancy (*p* < 0.001). SDMA (*p* < 0.01), L-arginine, and ADMA concentrations were higher in feed-restricted ewes than in controls. The L-arginine/ADMA ratio, an indicator of NO production by NOS, decreased towards term without differences between groups. The ADMA/SDMA ratio, an index of the ADMA degrading enzyme activity, was higher in controls than in feed-restricted ewes (*p* < 0.001). Obtained results show that circulating concentrations of L-arginine, of its metabolites, and the ratio between NO synthesis boosters and inhibitors are altered in energy-restricted ewes, and that these alterations are more marked in ewes carrying multiple fetuses.

## 1. Introduction

Nitric oxide (NO) is a key regulator of fetal homeostasis during pregnancy and it is considered the main vasodilator agent in the placenta, facilitating the maternal cardio-vascular changes, fetal development, and growth [1]. It has been identified in the syncytiotrophoblast and the fetoplacental vascular endothelium of mice where it contributes to lower fetoplacental vascular resistance and modulates other trophoblast functions such as implantation, differentiation, motility, invasion, and apoptosis [2].

The amino acid L-arginine, a substrate for the enzyme NO synthase (NOS), plays a key role in the production of NO, whereas the methylated arginine metabolites asymmetric dimethylarginine (ADMA) and, to a lesser extent, symmetric dimethylarginine (SDMA) are potent endogenous NOS inhibitors that reduce NO synthesis [3]. The L-arginine-ADMA axis strictly controls the production of NO in numerous physiological and pathological conditions, including pregnancy. For example, in humans, high ADMA concentrations have been consistently shown to correlate with cardiovascular diseases and preeclampsia [4]. The L-arginine to ADMA ratio can thus be used to assess altered NOS activity due to alterations of this axis [5].

L-homoarginine is a non-essential cationic amino acid that differs from L-arginine due to the presence of a methylene group (CH_2_). It represents an alternative substrate for NOS, and it can replace arginine as a substrate for the biosynthesis of NO [6,7]. In humans, high serum L-homoarginine concentrations have indeed been associated with enhanced endothelial function in the mother during pregnancy [8]. L-homoarginine concentrations have been shown to increase in normal pregnancy, probably due to the need for vasodilatation and enhanced endothelial function [8].

Considering the key role of NO in pregnancy, emerging therapeutic strategies involving NO precursors, NO donors, natural derivatives, or pharmacological modulators of the NO system seem promising for the treatment of intra-uterine growth restriction (IUGR). For the investigation of these therapeutic strategies, animal models of IUGR, such as the sheep, have been used [9]. However, in sheep, there is a lack of data on the concentrations of key regulators of NO production, such as ADMA, SDMA, and L-homoarginine, in normal and IUGR pregnancies. This information is of particular interest considering that in ewes carrying multiple fetuses, litter-size-dependent IUGR occurs naturally [10].

Therefore, the aim of this study was to investigate the blood concentrations of L-arginine, ADMA, SDMA, and L-homoarginine, modulating NO synthesis, in single, twin, and triplet pregnancies in ewes undergoing either normal nutrition or feeding restriction, an established model for IUGR [11]. In particular, the underlying hypothesis of the study was that IUGR induced by dietary energy-restriction could be associated with alterations in the circulating concentration of L-arginine, its metabolites, and the ratio between NO synthesis boosters and inhibitors, and that these alterations could be more severe in ewes gestating multiple fetuses. In addition, changes in circulating concentrations of L-arginine, ADMA, SDMA, and L-homoarginine were compared with those of total proteins and other amino acids not involved in the control of NO synthesis to rule out the possible direct effects of diet restriction on their concentrations.

## 2. Materials and Methods 

### 2.1. Ethics Statement

The experimental procedures with animals (sheep, *Ovis aries*) were approved by the Animal Care and Use Committee of the University of Sassari and of AGRIS, Italy (CIBASA 21.01.2014). All the experimental work was carried out at the facilities of AGRIS (Bonassai, Sardegna, Italy). These facilities meet the requirements of the European Union for Scientific Procedure Establishments. The experimental procedures followed ethical guidelines for care and use of animals for research (European Union Directive 2010/63/UE for animal experiments).

### 2.2. Animals and Experimental Procedure

We studied 59 adult (5.0 ± 3.0 years) and multiparous pregnant ewes (Sarda breed) from the experimental flock of AGRIS Sardegna (Italy). Pregnancy occurred after natural breeding following cycle synchronization with intravaginal pessaries impregnated with progestagens (20 mg of fluorogestone acetate, Chronogest^©^ CR; MSD-AH, Madison, NJ, USA) for 12 d plus a single i.m. injection of 200 IU of eCG (Folligon^©^; MSD-AH, Madison, NJ, USA), concurrent with pessary removal. The day of mating was considered day 0 for experimental purposes. From day 0 to day 24 of pregnancy, the ewes were permanently housed indoors and fed a standard pelleted concentrate and chopped ryegrass hay to fulfill the ewes’ daily requirements. At day 24 (16% of the total length of ovine pregnancy, estimated in a mean of 150 days), pregnancy diagnosis and initial fetal counts were conducted by transrectal ultrasonography, with a real-time B-mode scanner (Aloka SSD 500; Aloka Co., Tokyo, Japan) fitted with a 7.5 MHz linear-array probe. At the same time, the metabolic and hormonal profile of all the ewes was evaluated by assessing circulating concentrations of glucose, non-esterified fatty acids (NEFA), urea, total protein, albumin, alanine aminotransferase (ALT), aspartate aminotransferase (AST), lactate dehydrogenase (LDH), and progesterone. After scanning, the ewes were pair matched in two groups (control and feed-restricted) according to age, body weight (BW), and prolificacy (singleton, twins, and triplets). In sheep, placental growth is completed by day 100 of gestation and most of fetal growth occurs thereafter [12]. Taking this into account, from day 24 to day 100 of pregnancy, the ewes were fed the experimental diets (Table 1), which consisted of the same ryegrass hay and two different iso-proteic concentrates (high and low energy concentrates), whose formula was aimed at either fulfilling ewes’ energy requirements for maintenance and pregnancy (control group; BW 47.8 ± 5.2; *n* = 30, 14 singleton pregnancies, 12 twin pregnancies, and 4 triplet pregnancies) or only 45% of energy requirements (feed-restricted group; BW 48.1 ± 7.7; *n* = 29; 11 singleton pregnancies, 15 twin pregnancies, and 3 triplet pregnancies). The diets were formulated considering the average ewe’s live weights and the average fetal counts using the small ruminant nutrition system [13]. The iso-proteic concentrates were fed individually, while the hay was group-fed. Both diets were designed to meet ewes’ requirements for crude protein (CP), minerals, and vitamins and to ensure a sufficient content of rumen-effective neutral detergent fiber. Fresh water was available at all time. At day 45, fetal counts were reassessed as previously described. 

From day 24 to day 100, the diets were fed at the level of 0.9 kg/ewe per day (45% energy requirement of the feed-restricted group) and 1.5 fed kg/ewe per day (100% energy requirement of control group). These rations were ingested almost completely. From day 100 to lambing, experimental diets were adjusted fortnightly increasing the amount of the diet without changes in ingredients in order to account for the increasing requirements for pregnancy and weight changes. Group intakes were measured weighing the offer at each meal and the corresponding orts after each meal (concentrate) or after 24 h (hay). Average group intake of estimated ME and measured CP values are shown in Supplementary Table S1. From day 100 to lambing, the gap between the experimental diets was progressively reduced from 55% to 40% in order to reduce the risks of peri-natal mortality. This feeding strategy resulted in a gap of ME intake in the feed-restricted group as compared to control group falling from 57% to 37%, with an average of 53% (Supplementary Materials Table S1).

At parturition, pregnancy duration and number of lambs born were recorded and lamb live weight measured immediately after birth with an electronic scale. After parturition, ewes were transitioned to a common diet aimed at meeting maintenance and lactation requirements (expected milk yield: 1.5 kg of fat normalized milk per ewe per day). 

### 2.3. Blood Sampling

Blood samples were collected from all the ewes the day before intravaginal sponge insertion and thereafter every 30 days starting from day 24 until day 30 after parturition. Blood was sampled at 7:00 (before morning meal administration) using 10 mL vacuum collection tubes containing EDTA K2 (dipotassium ethylenediaminetetraacetic acid; Vacutainer Systems Europe; Becton Dickinson, MeylanCedex, Le Pont de Claix Cedex, France) to measure the circulating concentrations of arginine, ADMA, homoarginine, SDMA, alanine, cysteine, homocysteine, glycine, serine, taurine, and tryptophan. At day 24 and at day 140, to determine the metabolic and hormonal profiles, two additional blood samples were collected: One using 3 mL vacuum collection tubes containing lithium heparin and mono-iodioacetate (Vacutainer Systems Europe; Becton Dickinson, MeylanCedex, Le Pont de Claix Cedex, France) for the glucose assay, the other using 10 mL vacuum collection tubes containing EDTA K2 for the remaining analyses. The analytes quantified were alanine aminotransferase (ALT; U/L), albumin (g/dL), aspartate aminotransferase (AST; U/L), glucose (mg/dL), lactate dehydrogenase (LDH; U/L), non-esterified fatty acids (NEFA; mmol/L), urea (mg/dL), and progesterone (ng/mL). Immediately after collection, blood samples were cooled at 4 °C and centrifuged at 1500 *g* for 15 min. Plasma was removed and stored at −20 °C until assayed. All plasma samples were measured in duplicate. 

### 2.4. Assessment of Metabolic and Hormonal Profile

Glucose, NEFA, urea, albumin, ALT, AST, and LDH were measured using spectrophotometric commercial kits and BS-200 Mindray clinical chemistry analyzer. We used control I Normal (Wako) and control II Abnormal (Wako) as multi control for each measured parameter.

Glucose, NEFA and urea were measured using commercial kits and BS-200 Mindray clinical chemistry analyzer (Mindray, Nanshan, Shenzhen, China), as previously described [14]. Serum I Normal (Wako) and Serum II Abnormal (Wako) were used as multi control for each measured parameter. Glucose concentrations were determined in a single assay by liquid enzymatic colorimetric method (Glucose oxidase and Peroxidase) (Real Time Kits, Hagen Diagnostica srl, San Giovanni Valdarno, Italy), with a glucose standard of 100 mg/dL for calibration. Intra-assay CV values were 1.1%.

NEFA and urea concentrations were measured in multiple assays by enzymatic endpoint method (Diagnostic Systems GmbH, Holzheim, Germany) with a NEFA standard of 1 mmol/L and a urea standard of 50 mg/dL for calibration. NEFA intra-assay and interassay CV values were 1.07% and 0.98%, respectively. Urea intra-assay and interassay CV values were 1.7% and 1.6%, respectively.

Albumin, ALT, and AST were measured as previously described [15]. Albumin concentrations were measured in multiple assays by spectrophotometric endpoint method (Real Time Kits, Hagen Diagnostica srl, San Giovanni Valdarno, Italy), with an albumin standard (46.2 g/L) for calibration. Albumin in the sample reacts with bromocresol green in acid medium forming a colored complex measured at 630 nm. Intra-assay and inter-assay CV values were 1.36% and 1.52%, respectively.

ALT, AST, and LDH concentrations were measured in multiple assays by kinetics UV method (Real Time Kits, Hagen Diagnostica srl, San Giovanni Valdarno, Italy) using an ALT standard solution of 97.65 U/L, AST standard of 102 U/L and LH standard of 263 U/L for calibration. The catalytic concentration of ALT was determined from the rate of decrease of NADH, measured at 340 nm, by means of the lactate dehydrogenase coupled reaction. The catalytic concentration of AST was determined from the rate of decrease of NADH, measured at 340 nm, by means of the malatodehydrogenase coupled reaction. The catalytic concentration of LDH was determined from the rate of decrease of NADH, measured at 340 nm by lactate dehydrogenase reaction. ALT intra-assay and inter-assay CV values were 2.07% and 2.24%, respectively. AST intra-assay and inter-assay CV values were 2.42% and 2.29%, respectively. LDH intra-assay and inter-assay CV values were 2.41% and 4.49%, respectively.

Progesterone concentration was measured in duplicate using a commercial ELISA Kit (DRG Instruments GmbH, Marburg, Germany), which is a solid-phase ELISA, based on the principle of competitive binding. ELISA assay was performed using the Personal Lab Adaltis (Adaltis srl, Rome, Italy), which is a tool that performs automated ELISA protocols. All kit reagents, controls, and stored samples to be analyzed were thawed and warmed to 25 °C at the beginning of the test. The analytical sensitivity was 0.045 ng/mL and the intra-assay and inter-assay CV values were <10%.

### 2.5. Quantification of Circulating Concentrations of Arginine Metabolites, Other Amino Acids, and Total Proteins 

Arginine, ADMA, and SDMA were determined by capillary electrophoresis UV (Beckman Coulter Italia, Milan, Italy) detection as previously described [16]. The inadequate precision of the assay used for the analysis of ADMA may increase the chance of statistical type 2 errors in clinical studies with a severe underestimation of the strength of the association between ADMA and other biochemical or clinical variables [17]. For this reason, we used a capillary electrophoresis method with an inter-assay CV of between 2% and 3% for arginine, ADMA, and SDMA measurements. Taurine, serine, alanine, glycine, homoarginine, cysteine, and homocysteine were measured by capillary electrophoresis LIF (Beckman Coulter Italia, Milan, Italy) detection as previously described [18]. Inter-assay CV value for analytes was between 3% and 6%. Tryptophan was measured by capillary electrophoresis UV (Beckman Coulter Italia, Milan, Italy) detection as previously reported [19]. Inter-assay CV value was 7%. Plasma protein content was measured by Lowry’s method.

### 2.6. Homoarginine Detection by LC-MS/MS

In view of the high levels of homoarginine found during pregnancy, random samples have also been checked by LC-MS/MS to confirm method specificity [20]. For this purpose, plasma samples (200 μL) from 20 ewes in each group (10 single, 8 twin, and 2 triplet pregnancies—all sampling days) were spiked with 1 μL of the internal standard solution (d4-homoarginine) and vortexed. Tubes were then placed in a block heater for 5 min at 100 °C and subsequently cooled to room temperature, the clot was overlaid with 400 µL of ultrapure water. To displace the clot from the bottom of the vial, the containers were vortexed vigorously for 10 s. Dislodged clots were then placed in a block heater for 5 min at 100 °C. After vigorous vortex-mixing, samples were centrifuged at 17,000 *g* for 5 min. Supernatant (200 μL) was recovered and mixed with a 20 µL of phosphate buffer (100 mmol/L pH 7.0) and 40 µL of diethylpyrocarbonate (33 mmol/L). After vortex-mixing, tubes were left at room temperature for 1 min then analyzed by LC-MS/MS. Analyte separation was performed on a 100 mm × 4.6 mm Zorbax Eclipse Plus C18 3.5 µm column by using a mixture of an aqueous solution of 0.4% v/v formic acid and ACN (95:5) as a mobile phase, isocratically delivered at a flow-rate of 0.8 mL/min. Twenty microliters of sample were then injected in full loop mode by using a 20 µL sample loop. Column effluents were monitored by a mass spectrometer in a multiple reaction monitoring mode (MRM) with a run time of LC-MS/MS analysis (Waters Italia, Milan, Italy) of 10 min. The capillary voltage in the mass spectrometer (Waters Italia, Milan, Italy) was set at 2.5 kV, with a 150 and 500 °C ESI source and desolvation temperatures, Nitrogen, used as desolvation gas, was delivered at 600 L/h, while the argon in the collision cell was delivered at 0.5 mL/min. Mass detection was accomplished in positive ion mode by MRM of the precursor-product ion transitions m/z, 261.28→84, for homoarginine and m/z 265.28→88 for d4-homoarginine. Supplementary Materials Figure S1 shows a representative chromatogram of a sheep plasma sample at different withdrawal times. 

### 2.7. Statistical Analyses

Results are expressed as mean values (mean ± SEM) and the differences were considered to be statistically significant at *p* < 0.05. The distribution of variables in the study groups were assessed by the Kolmogorov–Smirnov test. The statistical differences between control and feed-restricted ewes for live weight, body condition score (BCS), duration of pregnancy, and lambs live weight were assessed using unpaired Student’s *t* test. These statistical analyses were performed using Minitab 17 Statistical Software (2010, Minitab, Inc., State College, PA, USA). Differences in metabolic and hormonal profile at 24 and 140 days of pregnancy between energy-restricted and control ewes were assessed using a One-way ANCOVA (analysis of covariance). A One-way ANCOVA was conducted to determine statistically significant differences between control and energy-restricted ewes, both during and after pregnancy, in amino acids concentrations controlling for pre-gestation values. Changes over time were assessed by ANCOVA for repeated measures using the Greenhouse–Geisser or Huynh–Feldt correction, as appropriate. The potential effects of the numbers of fetuses and the interaction with the treatment were evaluated and discarded when non-significant. ANCOVA was performed using SPSS (IBM Corp. Released 2016. IBM SPSS Statistics for Windows, Version 24.0. IBM Corp, Armonk, NY, USA).

## 3. Results

As shown in Supplementary Table S2, at day 24 of pregnancy, there were no significant differences between the two experimental groups in ewe’s live weight and BCS. As expected, control ewes carrying singleton and twins gained more weight during pregnancy compared to their energy-restricted counterparts and at day 140, live weight was significantly lower in energy-restricted compared to control ewes (*p* < 0.001). No difference was found in live weight of ewes carrying triplets between the two experimental groups. Differences in live weight between the two experimental groups were accompanied by differences in BCS only in ewes carrying twins (*p* < 0.001) and triplets (*p* = 0.07).

Delivery of singleton lambs occurred one day after in feed-restricted ewes compared to controls (*p* < 0.05), while no differences were observed in the duration of twin and triplet pregnancies between the two groups (Table 2). The total number of single, twin, and triplet lambs born in the two groups is shown in Table 3. Live weight at parturition was lower in singleton and twin lambs born from feed-restricted ewes when compared to controls, while there were no significant differences in triplet body weight between the two groups (Table 3). However, triplets showed a lower body weight when compared to singleton and twins from the same group (Table 3; *p* < 0.01). 

### 3.1. Metabolic and Hormonal Profile

As shown in Table 4, at day 24 and day 140 of pregnancy, there were no significant differences between experimental groups in the circulating concentrations of the analyzed metabolites, which were within the physiological ranges for this species [21]. In ewes gestating twin fetuses, progesterone concentrations were higher in controls than in feed-restricted ewes (*p* < 0.05; Table 4). 

### 3.2. Changes in Amino Acids and Arginine Metabolites during Pregnancy

**Figure 1:**
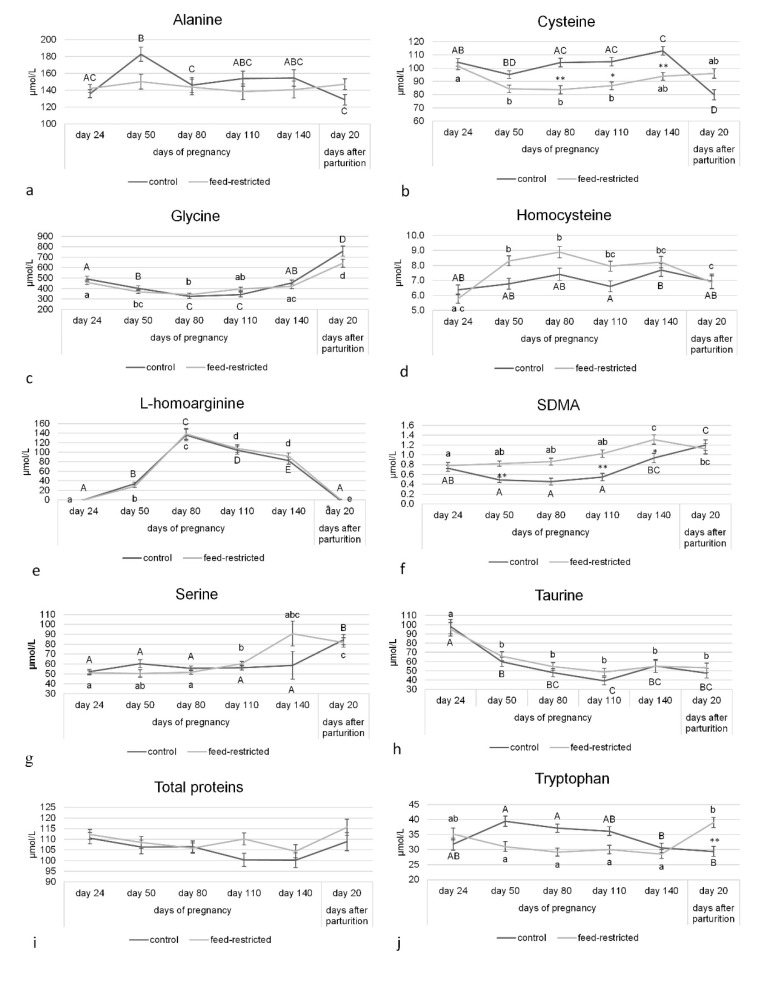
Circulating concentrations of arginine metabolites, other amino acids, and total proteins in control and feed-restricted ewes from day 24 of pregnancy to day 20 after parturition. (**a**) Alanine; (**b**) Cysteine; (**c**) Glycine: (**d**) homocysteine; (**e**) L-homoarginine; (**f**) SDMA; (**g**) serine; (**h**) Taurine; (**i**) Total protein; (**j**) Tryptophan. Asterisks indicate significant differences between control and feed-restricted ewes: ANCOVA * *p* < 0.05; ** *p* < 0.01. Different letters indicate significant differences within the same group: Upper-case letters for control group and lower-case letters for feed-restricted group.

No differences were observed in the circulating concentrations of alanine, glycine, serine, taurine, and total proteins between control and feed-restricted ewes from day 24 of pregnancy to day 20 after parturition (Figure 1). In both groups, glycine was the most abundant amino acid. By contrast, cysteine circulating concentrations were lower in feed-restricted ewes than in control ones from day 80 to day 140 of pregnancy (Figure 1b; *p* < 0.01), whereas mean concentration of homocysteine was higher in feed-restricted ewes than in controls (Figure 1d; *p* < 0.001). 

L-homoarginine increased markedly in both groups during pregnancy, from basal values of ≈ 1 µmol/L at day 24 to 136.1 ± 12.1 µmol/L in controls and 138.3 ± 11.5 µmol/L in feed-restricted ewes at day 80 (Figure 1e). At 20 days after parturition, its circulating concentrations, although markedly decreased and close to undetectable levels, were higher in feed-restricted ewes than in controls (*p* < 0.05). SDMA circulating concentrations were higher in feed-restricted ewes than in controls at days 50 and 110 of pregnancy (Figure 1f; *p* < 0.01). Finally, tryptophan plasma concentrations did not differ between the two groups from day 50 to day 140 of pregnancy (*p* > 0.05), but at 20 days after parturition they reached higher values in feed-restricted ewes than in controls (Figure 1j; *p* < 0.01). 

**Figure 2:**
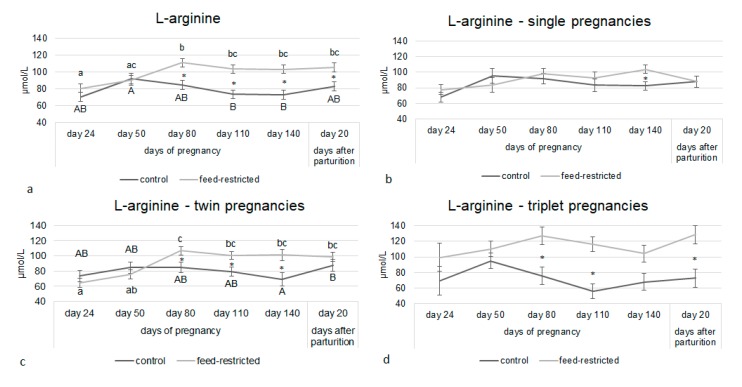
L-arginine circulating concentrations from day 24 of pregnancy to day 20 after parturition in control and feed-restricted ewes (**a**) carrying single fetuses (**b**), twins (**c**), and triplets (**d**). Asterisks indicate significant differences between control and feed-restricted ewes. Different letters indicate significant differences within the same group: Upper-case letters for control group and lower-case letters for feed-restricted group. ANCOVA * *p* < 0.05.

**Figure 3:**
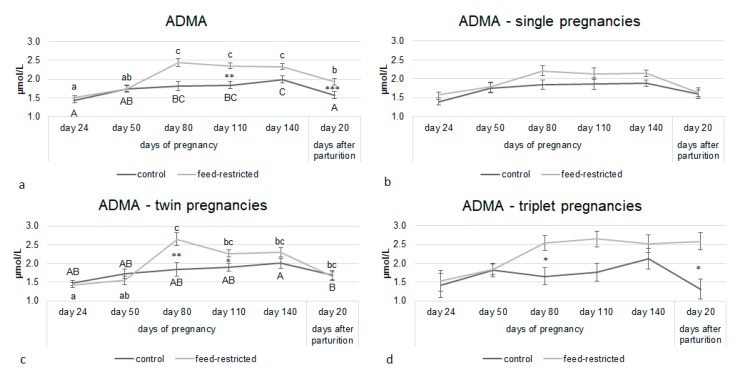
Asymmetric dimethylarginine (ADMA) circulating concentrations from day 24 of pregnancy to day 20 after parturition in control and feed-restricted ewes (**a**) carrying single fetuses (**b**), twins (**c**), and triplets (**d**). Asterisks indicate significant differences between control and feed-restricted ewes: ANCOVA * *p* < 0.05, ** *p* < 0.01; *** *p* < 0.001. Different letters indicate significant differences within the same group: Upper-case letters for control group and lower-case letters for feed-restricted group.

The number of fetuses carried and its interaction with the treatment had a significant effect on L-Arginine and ADMA plasma concentrations. L-arginine concentrations were higher in feed-restricted ewes than in controls from day 80 of pregnancy up to day 20 after parturition (Figure 2a). This difference was driven by ewes carrying twins and triplets (Figure 2c,d). A similar pattern was observed for ADMA, whose circulating concentrations were higher in feed-restricted ewes than in controls (Figure 3a) because of the effect caused by ewes carrying twins and triplets (Figure 3c,d).

**Figure 4:**
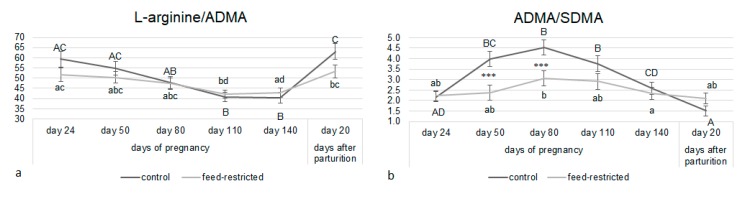
L-arginine/ADMA (**a**) and ADMA/symmetric dimethylarginine (SDMA) ratios (**b**) from day 24 of pregnancy to day 20 after parturition in control and feed-restricted ewes carrying single fetus, twins, and triplets. Asterisks indicate significant differences between control and feed-restricted ewes: ANCOVA *** *p* < 0.001. Different letters indicate significant differences within the same group: Upper-case letters for control group and lower-case letters for feed-restricted group.

The L-arginine/ADMA ratio, an indicator of NO production by NOS [22], decreased towards term without differences between the two groups (Figure 4a). The ADMA/SDMA ratio, an index of the ADMA degrading enzyme, dimethylarginine dimethylaminohydrolase activity [23], was higher in controls than feed-restricted ewes at day 50 and day 80 of pregnancy (Figure 4b; *p* < 0.001).

## 4. Discussion

As expected, nutrient restriction applied to pregnant ewes from day 24 of gestation to term led to IUGR in singleton and twin lambs. In triplet pregnancies, IUGR was induced by litter size, without differences in lamb body weight between controls and feed-restricted ewes. It is well known that litter-size-dependent IUGR occurs naturally in ewes carrying multiple fetuses [10]. However, we cannot rule out that group-feeding exacerbated this effect in this study. This could be related to the feeding management adopted, which was unable to cope with the variability of requirements within groups, particularly that related to the different types of pregnancy. Therefore, it is probable that, at least in the feed-restricted group, dietary energy deficit increased along with ewe’s litter size. As a matter of fact, BCS was significantly lower at the end of pregnancy only in feed-restricted ewes carrying twins and triplets.

In the present study, nutritionally induced IUGR was not associated with a direct deficiency in L-arginine, as reported by other authors [24,25], since circulating concentrations of arginine were higher in feed-restricted ewes than in controls. This result is of interest considering that concentrations of total proteins and of other amino acids showed no differences between the two experimental groups. Moreover, the difference in arginine concentrations between feed-restricted ewes and controls was dependent upon the number of fetuses carried, being enhanced in ewes carrying twins and triplets. Arginine in circulating blood is derived from diets and endogenous sources (protein degradation), however it can also be synthesized de novo in multiple species, including ruminants [26]. Therefore, the differences reported by other authors may be related to differences in diet, ewe’s metabolic requirements, number of fetuses carried, and duration of nutrient restriction. Other authors reported no changes in arginine concentrations in maternal jugular vein at day 113 of gestation in feed-restricted ewes carrying a single fetus compared to controls [27]. 

L-arginine, being a NO precursor, can enhance fetal weight in nutrient-restricted ewes by improving placental efficiency and nutrient availability for the fetuses [28]. Maternal L-arginine supplementation, in fact, has been effective in increasing fetal growth in several animal models of IUGR [25,29]. However, beneficial effects have not been universally shown [27]. We can speculate that in the present study, the increase in arginine plasma concentrations in feed-restricted ewes may represent a response mechanism to buffer the effects of feeding restriction on nutrient availability for the fetuses by improving placental efficiency. This response was more marked in ewes subjected to the greatest nutritional challenge, i.e., feed-restricted ewes carrying twins and triplets, where placental exchange area was limited by uterine capacity. Interestingly, arginine was not increased in control ewes carrying triplets with litter-size-dependent IUGR. 

Moreover, in feed-restricted ewes carrying twins and triplets, the higher arginine circulating concentrations were associated with higher concentrations of ADMA, its competitive inhibitor. As for arginine, ADMA was not increased in control ewes carrying triplets with litter-size-dependent IUGR. On the other hand, the significant increase in SDMA concentrations in feed-restricted ewes was not influenced by litter size. To the best of our knowledge, this is the first report on ADMA and SDMA circulating concentrations in ewes carrying single, twin and triplet pregnancies.

Circulating ADMA and SDMA derive exclusively from the arginine residues in certain proteins, which are methylated on their guanidine group by protein arginine methyltransferases [30]. Proteolysis of such NG-methylarginine proteins releases ADMA and/or SDMA, which can inhibit NOS activity in endothelial cells. ADMA and SDMA are cardiovascular risk factors/markers in adult humans [31]. ADMA’s cardiovascular risk is generally considered to be due to its inhibitory action of endothelial NOS (eNOS) activity, while SDMA is less potent than ADMA towards eNOS activity [3]. In pregnant women, elevated ADMA concentrations have been associated with endothelial dysfunction and the development of preeclampsia due to the reduced eNOS activity and NO availability in the cardiovascular system [4]. More recent studies suggest that maternal ADMA is a major repressor of the fetal growth by mechanisms independent of NO [32], with high plasma ADMA concentrations disrupting the placenta function through its interaction with insulin-like growth factors and/or their binding proteins (BP) and receptors [33]. However, while maternal plasma ADMA concentration is generally considered an important indicator of fetal growth restriction in women, underlying mechanisms are far from been elucidated. The role of SDMA is even harder to explain, being less potent than ADMA towards NOS activity. In the present study, ADMA and SDMA circulating concentrations were higher in feed-restricted ewes, adding evidences to the role of these arginine metabolites in the development of nutritionally induced IUGR in mammals. 

Since both arginine and ADMA increased in feed-restricted ewes, the arginine/ADMA ratio, a capacity measure of the arginine/NO pathway to generate NO, did not differ compared to control ewes. However, ADMA degrading enzyme activity was reduced in feed-restricted ewes compared to controls, as supported by the lower ADMA/SDMA ratio found. These observations suggest a possible alteration of the arginine/NO pathway in feed-restricted ewes. A previous study using the same sheep model of IUGR and performed in a subset of ewes used in the present study (control group; *n* = 12, six singleton pregnancies and six twin pregnancies; feed-restricted group; *n* = 12; four singleton pregnancies and eight twin pregnancies) supports this hypothesis [34]. Doppler ultrasonography performed at day 115 of pregnancy showed higher cerebro-umbilical ratios for systolic/diastolic ratio and resistance index in fetuses at the greatest challenge (twins in underfed pregnancies), which may indicate early stage of brain sparing [34]. 

Moreover, in feed-restricted ewes, homocysteine plasma concentrations were higher than in controls, and this increase was not influenced by litter size. Homocysteine increases ADMA level by inhibiting dimethylaminohydrolase (DDAH), which hydrolyzes and degrades ADMA [35], and leads to endothelial dysfunction [36]. Several studies have reported that homocysteine concentration increases in women with hypertensive disorders of pregnancy [35,36,37]. Hyperhomocysteinemia is also known to cause endothelial dysfunction, independent of induction of ADMA [38]. Thus, the observed increase in ADMA circulating concentration in feed-restricted ewes carrying multiple fetuses might be secondary to a decrease in ADMA degrading enzyme activity caused, at least partly, by hyperhomocysteinemia. 

The present study also reported a more than a 100-fold increase in L-homoarginine circulating concentrations during pregnancy both in feed-restricted and control ewes. Due to this remarkable increase and in view of the lack of information on the concentration of this metabolite in pregnant ewes, random samples were also checked for homoarginine concentration by LC-MS/MS. Obtained results confirmed the concentrations found by CE-LIF method.

The maximal binding rate of L-arginine to NOS is twice that of L-homoarginine [39], and thus L-arginine is considered the more significant substrate in the regulation of the NO pathway. L-homoarginine acts as an alternative substrate for the synthesis of nitric oxide, but it also increases the intracellular L-arginine concentration [8] and inhibits the enzyme arginase from competing with NOS for L-arginine [40]. A previous study [8] measured homoarginine, arginine, and ADMA in pregnant women and non-pregnant controls and found that homoarginine plays a direct role in NO upregulation during pregnancy. Wu [41] reported that the endogenous production of L-arginine is inadequate for the pregnant females. The significant increase in L-homoarginine concentrations during pregnancy may thus compensate for the insufficient endogenous production of L-arginine [42]. Unfortunately, the comprehensive physiological roles of the L-homoarginine during pregnancy still need to be elucidated. Results of the present study show original data on the significant increase in L-homoarginine circulating level in pregnant ewes and further support the importance of homoarginine in the adaptive changes of pregnancy in mammals. 

Taken together, obtained results show that circulating concentrations of L-arginine, ADMA, SDMA, and the ratio between NO synthesis boosters and inhibitors are altered in energy-restricted ewes, and that these alterations are more marked in ewes carrying multiple fetuses. In addition, reported results suggest different underlying mechanisms for the development of litter-size-dependent and nutritionallyinduced IUGR, which involve the arginine/NO pathway.

## Figures and Tables

**Table 1:** Ingredients (g/kg), diet chemical composition (g/kg), and estimated metabolizable energy (Mcal/kg).

Ingredients	Feed-Restricted Diet	Control Diet
	50% Energy Requirement	100% Energy Requirement
Ryegrass hay	316	281
Concentrate Low ^1^	684	-
Concentrate High ^2^	-	719
Dry matter	902	886
Ash	99	85
Ether Extract	22	28
Crude protein	133	121
Neutral Detergent Fibre	465	393
Acid Detergent Fibre	300	218
Acid Detergent Lignin	83	30
Metabolizable energy	1.442	1.926

^1^ Based on (g/kg) dehydrated lucerne (607), cocoa hulls (200), wheat middlings (106), sugarcane molasses (52), and inclusive (per kg fresh weight) of Ca 11.4 g; P 0.45 g; Vit. A 16,000 IU; Vit. D_3_ 3000 IU; Vit. E 50 mg; manganic oxide 77 mg; zinc oxide 186 mg; cobalt carbonate 0.36 mg; ferrous carbonate 124 mg; anhydrous calcium iodate 3.9 mg; sodium selenite 0.88 mg, arginine 56 mg. ^2^ Based on (g/kg) wheat bran (250), dehydrated lucerne (154), wheat meal (150), soybean hulls (100), corn gluten meal (63), sugarcane molasses (60), dehydrated sugarbeet pulp (60), cocoa hulls (50) maize grain (41), sunflower meal (39), and inclusive (per kg fresh weight) of Ca 11.4 g; P 0.45 g; Vit. A 16,000 IU; Vit. D_3_ 3000 IU; Vit. E 50 mg; manganic oxide 77 mg; zinc oxide 186 mg; cobalt carbonate 0.36 mg; ferrous carbonate 124 mg; anhydrous calcium iodate 3.9 mg; sodium selenite 0.88 mg, arginine 67 mg.

**Table 2:** Duration of pregnancy in feed-restricted and control ewes (mean ± SEM).

Pregnancy (Days)	Group		
	Control (*n*)	Feed-Restricted (*n*)	*p* Value
Mean values	149.5 ± 0.3 (30)	150.5 ± 0.4 (29)	0.031
Singleton	149.4 ± 0.4 (14)	150.8 ± 0.5 (11)	0.049
Twins	149.4 ± 0.6 (12)	150.7 ± 0.5 (15)	0.109
Triplets	149.7 ± 1.2 (4)	149.0 ± 0.1 (3)	0.699

**Table 3:** Lamb body weight at parturition in feed-restricted and control ewes (mean ± SEM).

Lamb Weight (kg)	Group		
	Control (*n*)	Feed-Restricted (*n*)	*p* Value
Mean values	3.27 ± 0.1 (47)	2.57 ± 0.1 (52)	0.001
Singleton	3.72 ± 0.2 (14) ^a^	2.93 ± 0.2 (11) ^a^	0.007
Twins	3.30 ± 0.1 (24) ^a^	2.59 ± 0.1 (30) ^a^	0.001
Triplets	2.50 ± 0.6 (9) ^b^	2.17 ± 0.4 (11 *) ^b^	0.098

* One lamb died at parturition and was excluded from the study. ^a,b^ Different letters indicate differences within the same group: *t*-test *p* < 0.01.

**Table 4:** Circulating concentrations of metabolites and progesterone at day 24 and day 140 of pregnancy in control (14 singleton pregnancies, 12 twin pregnancies, and 4 triplet pregnancies) and in feed-restricted ewes (11 singleton pregnancies, 15 twin pregnancies, and 3 triplet pregnancies). T = treatment; D = day; ALT = alanine aminotransferase; AST = aspartate aminotransferase; LDH = lactate dehydrogenase; NEFA = non-esterified fatty acids.

Variables	Litter Size	Treatments (T)		Day of Pregnancy		*p*-Values		
		Controls	Feed-Restr.	24	140	T	D	T × D
ALT (U/L)	Singletons	25.9 ± 1.7	24.8 ± 2.0	26.2 ± 1.3	24.5 ± 1.6	0.696	0.175	0.526
	Twins	22.1 ± 1.4	25.0 ± 1.3	24.9 ± 1.4	22.1 ± 0.9	0.137	0.071	0.795
	Triplets	20.6 ± 2.2	24.7 ± 2.2	25.6 ± 1.6	19.7 ± 1.7	0.246	0.002	0.810
Albumin (g/dL)	Singletons	3.5 ± 0.1	3.4 ± 0.1	3.5 ± 0.1	3.4 ± 0.1	0.626	0.325	0.224
	Twins	3.6 ± 0.1	3.5 ± 0.1	3.5 ± 0.1	3.5 ± 0.1	0.516	0.406	0.681
	Triplets	3.5 ± 0.1	3.4 ± 0.1	3.6 ± 0.1	3.3 ± 0.1	0.681	0.078	0.994
AST (U/L)	Singletons	161.6 ± 19.0	141.5 ± 20.7	171.0 ± 21.9	132.1 ± 10.2	0.483	0.060	0.420
	Twins	144.8 ± 15.3	138.9 ± 14.1	155.7 ± 11.2	128.0 ± 11.0	0.781	0.002	0.436
	Triplets	125.1 ± 15.4	108.3 ± 15.4	121.0 ± 7.0	112.5 ± 15.4	0.470	0.419	0.940
Glucose (mg/dL)	Singletons	82.3 ± 2.6	76.8 ± 2.9	81.9 ± 2.2	77.3 ± 2.0	0.167	0.014	0.507
	Twins	78.0 ± 1.9	76.7 ± 1.7	81.4 ± 1.0	73.3 ± 2.1	0.606	0.001	0.484
	Triplets	72.9 ± 4.4	66.9 ± 4.4	77.0 ± 3.4	62.8 ± 4.5	0.377	0.030	0.088
LDH (U/L)	Singletons	556.0 ± 42.2	531.1 ± 49.9	577.0 ± 50.3	510.0 ± 26.2	0.707	0.163	0.234
	Twins	707.2 ± 122.0	549.9 ± 109.1	746.6 ± 163.4	510.5 ± 26.5	0.346	0.170	0.390
	Triplets	535.5 ± 44.6	466.7 ± 44.6	490.7 ± 12.6	511.5 ± 52.9	0.317	0.653	0.360
Progesterone (ng/mL)	Singletons	12.6 ± 1.4	9.3 ± 1.6	6.8 ± 0.8	15.1 ± 1.5	0.141	0.000	0.099
	Twins	23.5 ± 2.3	15.6 ± 1.6	9.0 ± 1.3	30.1 ± 2.6	0.011	0.000	0.205
	Triplets	25.1 ± 8.5	35.2 ± 7.4	11.9 ± 0.9	48.5 ± 11.2	0.411	0.023	0.319
NEFA (mmol/L)	Singletons	0.2 ± 0.0	0.2 ± 0.0	0.3 ± 0.0	0.1 ± 0.0	0.549	0.000	0.554
	Twins	0.3 ± 0.0	0.3 ± 0.0	0.4 ± 0.1	0.3 ± 0.0	0.750	0.055	0.738
	Triplets	0.3 ± 0.1	0.2 ± 0.1	0.3 ± 0.0	0.2 ± 0.1	0.180	0.332	0.619
Urea (mg/dL)	Singletons	29.7 ± 1.5	31.4 ± 1.7	31.7 ± 1.6	29.5 ± 1.0	0.445	0.154	0.622
	Twins	26.7 ± 1.3	29.3 ± 1.2	29.3 ± 1.2	26.7 ± 1.0	0.166	0.057	0.161
	Triplets	25.9 ± 2.2	29.1 ± 2.2	29.2 ± 2.6	25.7 ± 1.7	0.333	0.307	0.139

## Supplementary Materials

The following are available online at https://www.mdpi.com/2076-2615/10/1/65/s1, Figure S1: Representative chromatogram of a real sheep plasma sample at different withdrawn times (d24 and d80). Inset shows MS/MS spectra of derivatized homoarginine. Table S1: Intake of metabolizable energy and crude protein in control (14 singleton pregnancies, 12 twin pregnancies and 4 triplet pregnancies) and in feed-restricted pregnant ewes (11 singleton pregnancies, 15 twin pregnancies and 3 triplet pregnancies), Table S2: Live weights and body condition scores (BCS) at day 24 and day 140 of pregnancy in control (14 singleton pregnancies, 12 twin pregnancies and 4 triplet pregnancies) and in feed-restricted ewes (11 singleton pregnancies, 15 twin pregnancies and 3 triplet pregnancies).
