# Supplementary material for: Circulating Concentrations of Key Regulators of Nitric Oxide Production in Undernourished Sheep Carrying Single and Multiple Fetuses
Source: Animals (Basel). 2019 Dec 30;10(1):65. doi: 10.3390/ani10010065 (PMC7023428; doi:10.3390/ani10010065)
Supplement: Supplementary file 1 [file animals-10-00065-s001.pdf]

animals

MDPI

Circulating Concentrations of Key Regulators of Nitric Oxide Production in Undernourished Sheep Carrying Single and Multiple Fetuses

Fiammetta Berlinguer [1,*], Cristian Porcu [1], Giovanni Molle [2], Andrea Cabiddu [2], Maria Dattena [2], Marilia Gallus [2], Valeria Pasciu [1], Sara Succu [1], Francesca D. Sotgiu [1], Panagiotis Paliogiannis [3], Salvatore Sotgia [3], Arduino A Mangoni [4], Antonio Gonzalez-Bulnes [5], Ciriaco Carru [3] and Angelo Zinellu [3]

Table S1. Intake of metabolizable energy and crude protein in control (14 singleton pregnancies, 12 twin pregnancies and 4 triplet pregnancies) and in feed-restricted pregnant ewes (11 singleton pregnancies, 15 twin pregnancies and 3 triplet pregnancies).

Days From Mating	Intake of Metabolizable Energy (Mcal/ewe day)		Intake of Crude Protein (g/ewe day)	
	Control	Feed-restricted	Control	Feed-restricted
24–100	2.96	1.26	186	116
101–114	3.64	1.59	229	146
115–128	3.56	1.98	224	182
129–142	3.49	2.20	219	203
Overall Mean	3.17	1.50	199	138

Table S2. Live weights and body condition scores (BCS) at d 24 and d 140 of pregnancy in control (14 singleton pregnancies, 12 twin pregnancies and 4 triplet pregnancies) and in feed-restricted ewes (11 singleton pregnancies, 15 twin pregnancies and 3 triplet pregnancies).

**Variables**	**Type of Pregnancy**	**Day 24 of pregnancy**			**Day 140 of pregnancy**			**Control d 24 vs d 140**	**Feed-restricted d 24 vs d 140**
		**Controls**	**Feed-restricted**	***p* value**	**Controls**	**Feed-restricted**	***p* value**	*p* value	*p* value
Body weight (kg)	Singletons	48.0 ± 5.9	45.2 ± 9.0	0.38	60.1 ± 6.5	47.5 ± 7.6	$p < 0.001$	$p < 0.05$	0.53
	Twins	48.6 ± 4.3	49.0 ± 7.0	0.85	62.2 ± 6.1	54.1 ± 6.6	$p < 0.005$	$p < 0.001$	$p < 0.05$
	Triplets	43.7 ± 4.6	52.7 ± 3.4	0.06	58.0 ± 7.9	57.3 ± 4.9	0.90	0.07	0.17
BCS	Singletons	2.64 ± 0.25	2.65 ± 0.27	0.98	2.87 ± 0.27	2.46 ± 0.39	0.60	$p < 0.001$	0.19
	Twins	2.75 ± 0.20	2.69 ± 0.24	0.49	2.77 ± 0.17	2.40 ± 0.15	$p < 0.001$	0.71	$p < 0.01$
	Triplets	2.76 ± 0.12	2.64 ± 0.32	0.54	2.63 ± 0.26	2.13 ± 0.27	0.07	0.49	0.06

\

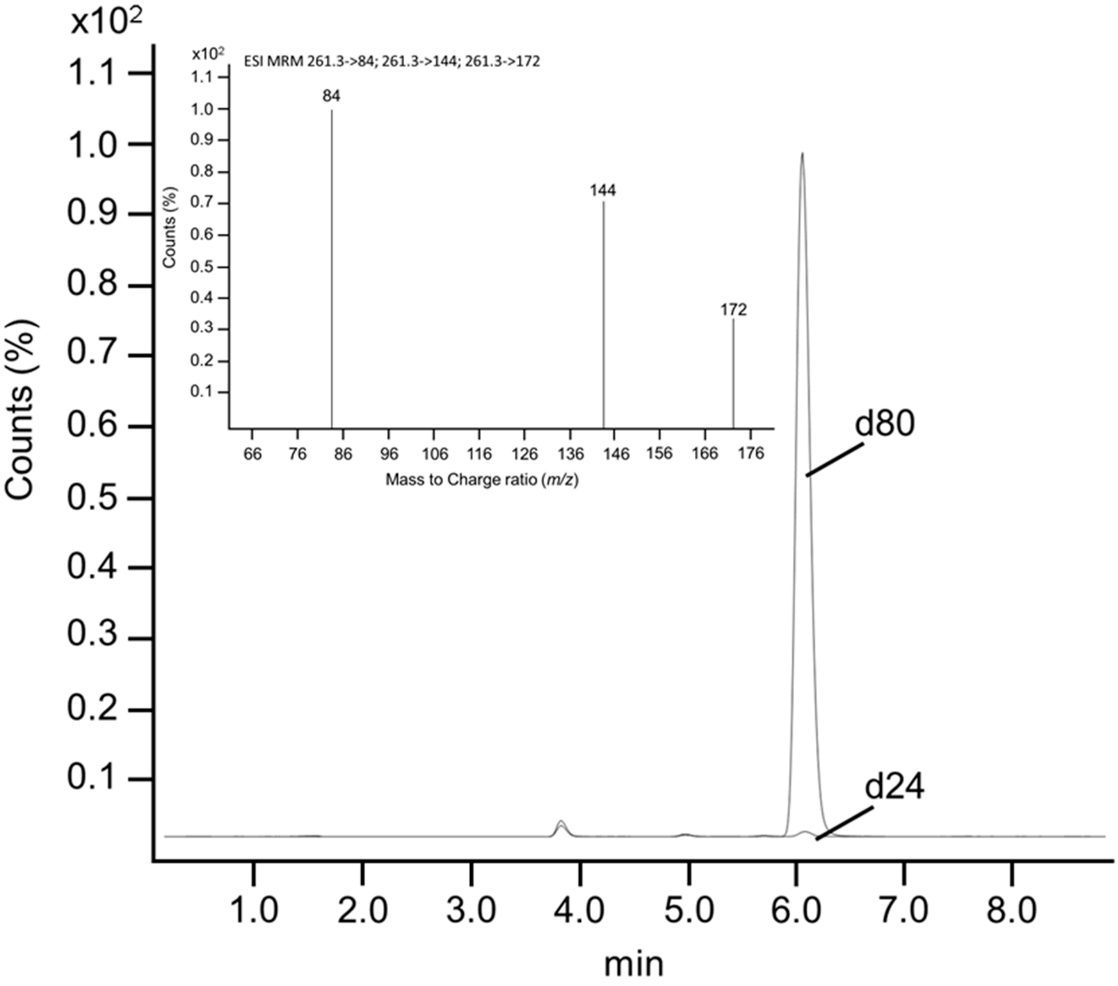

Figure S1. Representative chromatogram of a real sheep plasma sample at different withdrawn times (d24 and d80). Inset shows MS/MS spectra of derivatized homoarginine.
